# Increase in Grasp Force Reflects a Desire to Improve Movement Precision

**DOI:** 10.1523/ENEURO.0095-19.2019

**Published:** 2019-07-19

**Authors:** A. Takagi, H. Kambara, Y. Koike

**Affiliations:** 1 Tokyo Institute of Technology, Institute of Innovative Research, Yokohama 226-8503, Japan; 2 Precursory Research for Embryonic Science and Technology (PRESTO), Japan Science and Technology Agency (JST), Kawaguchi, Saitama 332-0012, Japan

**Keywords:** grasp force, movement precision

## Abstract

Grasping is an action engraved in the human genome, enabling newborn infants to hang from a monkey-bar immediately after birth. The grasp force provides rich information about the brain’s control of arm movements. In this study, we tested the hypothesis that the grasp force increases to improve the hand’s movement precision during reaching. In two reaching experiments, subjects increased grasp force to suppress movement imprecision that arose from both self-generated motor noise and from an unpredictable environment. Furthermore, the grasp force did not increase constantly, but increased specifically along the movement where the hand’s deviation was greatest. The increased grasp was premeditated and was not a reaction to environmental forces, suggesting that the central nervous system has a predictive, state-dependent model of movement precision during reaching. The grasp force provides a high temporal resolution and calibration-less estimate of movement precision adaptation.

## Significance Statement

Humans use their hands on a daily basis to interact with the environment. Many tasks require the hand’s movement to be precise. Standard measures of movement precision resort to measuring the stiffness of the arm, which is notoriously difficult to measure during motion. We show that the power grasp force is correlated with movement precision, and that it provides a real-time measure of movement precision adaptation. Furthermore, the grasp force measure reveals that the brain has a state-dependent adaptation of movement precision, such that it increased grasped force in locations where the hand’s deviation was greatest.

## Introduction

Grasping with the hand is a fundamental motor action in humans that can be evoked in infants (McGraw, 1940), alongside the traction response, where the passive stretching of the shoulder abductors and the arm’s flexors cause the fingers, elbow, and shoulder flexors to flex in synergistic response (Twitchell, 1965). As infants mature, their arm movements become smoother and more precise (Thelen et al., 1996). As adults, humans rarely make mistakes when moving the arm, like when reaching to grab a mug. However, some skilled movements that require precision are difficult even for adults.

Taking hammering as an example, the hammer must strike the nail head precisely, which is challenging due to self-generated motor noise (Selen et al., 2009). The head of the hammer must remain flat against the nail during contact, which is difficult as unpredictable contact forces can destabilize the hammer (Ganesh et al., 2012). Failure at such tasks occurs when the hand’s movement is perturbed unpredictably because the central nervous system (CNS) uses delayed sensory feedback to correct its movement (Pew et al., 1967). Thus, both unpredictable self-generated motor noise and environmental interactions result in reduced movement precision that cannot be corrected immediately by the CNS. It should be noted that precision is different from accuracy, as precision relates to variance whereas accuracy refers to bias.

A vast literature exists on how humans adapt to a force field that perturbs the accuracy of the hand’s motion when reaching toward a point target (Shadmehr et al., 2010). The pioneering study of Shadmehr and Mussa-Ivaldi (1994) revealed the ability of the CNS to learn to move in a velocity-dependent force field. Before the introduction of the force field, the hand moves from one point to another in a straight but somewhat curved trajectory. When the force field is introduced, the hand’s trajectory curves outward and causes the subject to miss the intended target. Movement accuracy is regained by learning the dynamics of the force field and countering the force field’s effects on the hand via appropriate forces produced by the hand (Conditt et al., 1997).

To recover movement accuracy in the force field, a forward model of the force field’s dynamics is learned by the CNS. However, if the external forces are unpredictable and cause movement imprecision, the CNS uses a different strategy of coactivating the muscle pairs in the arm to increase its stiffness, which reduces the impact of unpredictable forces on the movement of the arm (Hogan, 1984; Burdet et al., 2001; Wong et al., 2009). Thus, the CNS’ adaptation of movement precision in the presence of unpredictable external forces has been estimated by measuring the stiffness or the cocontraction of the arm (Franklin et al., 2008).

A recent study reported another method of measuring the CNS’ adaptation to unpredictable forces. The authors of this study measured increases in the pinch grip force when subjects reached in an unpredictable force field (Hadjiosif and Smith, 2015). They found a positive correlation between the pinch grip force and the variability of the external forces. One issue with the pinch grip methodology is the strong coupling between the pinch grip force and the load force, which may confound the interpretation of the data. Furthermore, Hadjiosif and Smith (2015) did not test whether subjects increase pinch grip force when increased movement precision is desired in the absence of external forces.

In this study, we hypothesize that the power grasp force positively correlates with the desire to increase movement precision. We test two predictions based on this hypothesis in two experiments. First, we test the hypothesis that the grasp force increases when subjects want to improve movement precision. This hypothesis is tested by asking subjects to keep their hand within a wide or narrow visual track while reaching toward a target, and measuring the changes in the grasp force. Second, we hypothesize that changes in grasp force reflect a desire to improve movement precision, and do not reflect the actual movement precision per se. This second hypothesis is tested by asking subjects to reach in a diverging force field that pushed their hand laterally from the center line. As subjects were unaware of when the force field would activate, we predicted that the grasp force would not increase in response to the force field on the first trial. We also predicted that in catch trials, where the force field was unexpectedly turned off, the grasp force would not decrease although the actual movement precision was high.

## Materials and Methods

Ten male subjects, who all gave their informed consent, participated in the study. The experiment was conducted in accordance with the Declaration of Helsinki, and the study was approved by the Ethical Review Board for epidemiological Studies at the Tokyo Institute of Technology.

**Figure 1.**
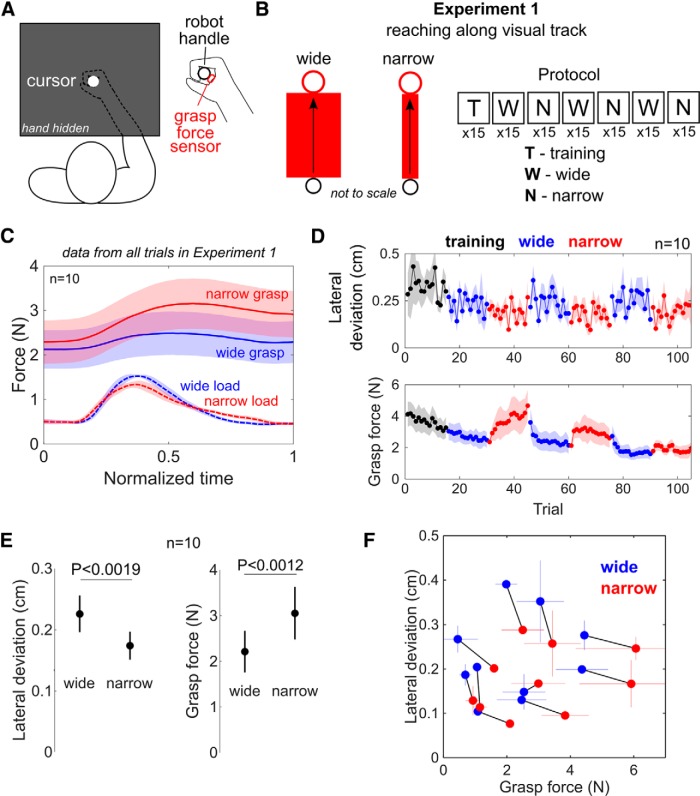
Experimental setup, protocol, and results from the first experiment where subjects reached along a visual track ***A***, Subjects held the handle of a robotic manipulandum, where the position of their arm was hidden behind a film mirror. Subjects received visual feedback of the position of their hand as a cursor on the monitor such that it could be seen above the hand. The elbow was supported such that the hand, elbow, and shoulder were level along a horizontal plane. A force sensor was placed between the palm of the hand and the handle to measure the grasp force. ***B***, In the first experiment of reaching along a visual track, subjects were presented with two visual feedback conditions showing either a wide or a narrow track. Subjects were instructed to keep their cursor inside the track and reach the circular target at the end of the track. Subjects first made reaching movements without a visual track in a training block, after which they experienced wide and narrow blocks in consecutive sequence for three repetitions per condition. ***C***, The group mean grasp force (solid traces) and load force (dotted traces) are plotted as a function of normalized time, where the shaded area is 1 SE. The data were separated into the wide (blue) and the narrow (red) conditions. The Pearson correlation coefficient between the grasp and load forces was not significantly different from zero. Thus, no significant correlation between grasp and load force was observed when reaching along a visual track. ***D***, The lateral deviation and the grasp force of the population mean and SE are plotted as a function of trials with the plot color indicating the training trials (black), wide track (blue) and narrow track (red) conditions. ***E***, The mean lateral deviation and the mean grasp force from the population is shown for the wide and narrow conditions. The lateral deviation was lower and the grasp force was higher when the visual track was narrow. ***F***, The lateral deviation is plotted as a function of the grasp for every subject in the wide and narrow conditions, with a black line connecting the data from the same subject. The thin blue and red lines show standard deviation from the linear mixed effects model fits. An increase in grasp force was observed to subsequently reduce the lateral deviation.

The subjects were seated facing the KINARM planar robotic manipulandum from BKIN Technologies (Fig. 1). Subjects held onto the KINARM interface via a handle that was affixed with a three-axis force sensor (Tec Gihan) to measure the grasp force from the palm of the hand during reaching movements. An Edero Armon arm support was used under the elbow to support the arm’s weight when using the robotic interface. Visual feedback was provided on a monitor that was placed upside-down such that subjects viewed a reflection of the monitor on a thin film mirror placed above the hand, which obscured it from view. The position of the hand was visible as a white circular cursor during both the visual track and the divergent force field experiments.

### Visual track reaching experiment

Subjects moved their hand to reach a target of radius 2 cm placed 25 cm away from their initial starting position. Subjects were instructed to prevent the cursor from deviating off a red visual track that was displayed between the initial and target positions (Fig. 1*B*).

The experimental structure consisted of seven blocks where each block contained 15 trials. The first block consisted of training trials, where the red region was absent. Blocks 2, 4, and 6 were the “wide” condition that demanded low movement accuracy blocks as the visual track was ±4 cm wide. On the other hand, the “narrow” condition tested in blocks 3, 5, and 7 required high movement accuracy due to a narrow visual track with a width of ±0.4 cm. The cursor in this experiment had a diameter of 0.4 cm. Feedback of the movement duration was provided to the subject on a trial-by-trial basis. Movements that were faster than 900 ms and longer than 1100 ms triggered a feedback message of “fast” or “slow,” respectively, to ensure that subjects reached with comparable movement speeds in both the wide and narrow conditions. No feedback was given of the lateral deviation after the trial.

As we observed a linear relationship between both the lateral deviation and the grasp force as a function of trials, we fitted these data from the visual track experiment using the linear mixed-effects model of the form

(1) $$Y=\beta_{0s}+\beta_{1s}C+\beta_{2s}T+\beta_{3s}\left(T\cdot C\right)+\varepsilon_{s},$$

where the response *Y* is either the vector of data from grasp force *F_G_* or from the lateral deviation *x_LD_*, *T* is the trial number, *C* is the visual track condition (narrow or wide), $\beta_{0s}$ is the intercept, $\beta_{1s}$ to $\beta_{3s}$ are the parameters for each predictor, and $\varepsilon_{s}$ is the unexplained variance of the response *Y* for each subject *s*.

A likelihood ratio test was employed to examine the significance of the condition parameter *C* on explaining the data. If deemed significant, this implied that the width of the visual track had a significant impact on the grasp force and the lateral deviation.

If the condition was deemed to significantly influence the grasp force or the lateral deviation, a one-sample *t* test was conducted on the data, which was grouped separately for the wide and narrow conditions. This enabled us to test our hypothesis of whether the grasp force increased when reaching along a narrow visual track and whether a reduction in lateral deviation was observed in the narrow track. However, these tests alone were not sufficient to establish a relationship between the grasp force and the lateral deviation as they only examine the effect on a group level. We examined how each subject’s lateral deviation changed as a function of the change in their grasp force. Here, a non-parametric sign test was employed as these data were observed to violate normality. The normality of all datasets was tested using an Anderson–Darling test before *post hoc* testing.

### Divergent force field experiment

**Figure 2.**
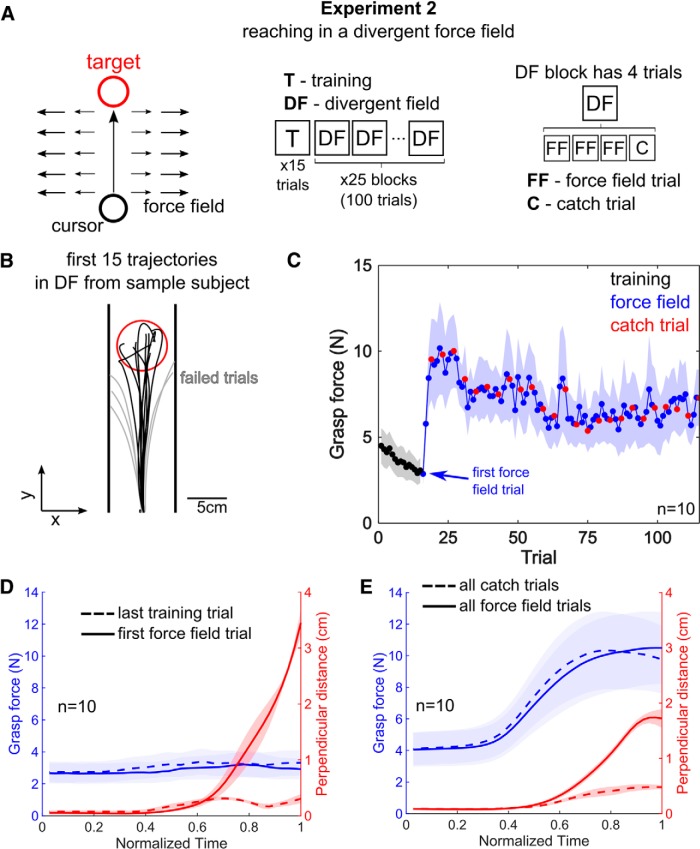
The divergent force field reaching experiment supports the hypothesis that the grasp force increases when better movement precision is desired, and not when the actual movement precision changes. ***A***, A schematic of the experiment and its protocol. The force field pushed the hand away laterally if it deviated from the line that connected the start and the target positions. To succeed, a subject must reach as straight as possible with minimum lateral deviation. Subjects experienced 15 training trials in the null field conditions, after which they experienced 100 force field trials. Of these 100 trials, every fourth trial was a catch trial where the force field was switched off. ***B***, The first 15 trajectories in the force field trials from a sample subject are shown, where the gray trajectories show failed trials where their hand hit the safety margins placed 4 cm to the left and right of the center line. A trial was successful when subjects stopped inside the red target. ***C***, The mean grasp force from all subjects, averaged over each trial, is plotted as a function of trials. In training trials (black), the grasp force continually declines. The grasp force in the first force field trial is similar to the level observed in training trials, but begins to increase until it peaks at approximately the fourth force field trial. Although the grasp force declines during the force field trials, it never reaches the same level as the training trials. Furthermore, the grasp force in catch trials is indistinguishable from the force field trials, revealing that the grasp is not a reaction to the forces from the force field, but is premeditated. ***D***, The group mean grasp force from the last training trial (dashed blue trace) and the first force field trial (solid blue trace) are plotted as a function of normalized time. On the same figure, the group mean perpendicular distance from the last training trial (dashed red trace) and the first force field trial (solid red trace) are plotted as a function of normalized time. Although the perpendicular distance increased dramatically due to the force field, the grasp force remained constant. ***E***, The group mean grasp force (blue traces) and the group mean perpendicular distance (red traces) from all catch trials (dashed blue trace) and all force field trials (solid blue trace) are plotted as a function of normalized time. The grasp force was similar between the catch trials and the force field trials, although the perpendicular distance was smaller in catch trials where the force field was switched off.

The same 10 subjects that took part in the first experiment participated in the divergent force field task (Fig. 2*A*). Subjects were instructed to reach a target 20 cm away from the starting position. The diameter of the cursor was 1 cm in this experiment. Feedback was provided on a trial-by-trial basis about the duration of the movement, which had to be between 500 and 600 ms. Each participant practiced a standard reaching task for 15 training trials, after which they experienced 25 divergent field blocks. The divergent force field was designed to amplify lateral reaching errors by applying the following force to the hand,

(2) $$F_{x}=kx\,\;\mathrm{for}\left|x\right|<0.04m\text{,}$$

where the stiffness k=400N/m and *x* is the lateral position of the hand such that the initial and target positions are at x=0. Each divergent field block was composed of four trials where the last trial was a catch trial where the force field was switched off, i.e., $F_{x}=0$. The catch trials tested whether subjects were simply grasping the handle as a reaction to the forces from the robot, or were increasing their grasp force to improve their movement precision. If the hand’s lateral deviation was greater than ±4 cm in a force field trial, the force field was switched off and the subjects were shown a “failed” trial message.

*Post hoc* one-sample *t* tests were conducted to examine the difference between the last training trial and the first force field trial, and the difference between the catch trials and the force field trials. If the grasp force is different in either of these cases, our hypothesis must be rejected.

## Results

### Experiment 1: reaching along a wide or narrow visual track

In the first experiment, subjects had to make point-to-point reaching movements toward a circular target of radius 2 cm that was placed 25 cm away from the initial starting position. Subjects were instructed to prevent the cursor from deviating off a red visual track that was displayed between the initial and target positions (Fig. 1*B*).

The literature reports that, with a pinch grip, significant correlation is observed between the pinch grip force and the load force (Flanagan and Wing, 1997). Such a correlation could undermine our study as the grasp force may simply reflect the load experienced by the hand during reaching. The grasp force and the load force are plotted as a function of normalized time in Figure 1*C*. The data from every trial’s whole movement were selected for this analysis, where the start of the trial was when the target appeared, and the end was when the hand stopped inside the target. We calculated the Pearson correlation coefficient in all trials between the grasp force and the load force supplied by the subject against the robotic interface. If the load force has a significant impact on the grasp force measurement, this must be taken into account in subsequent analysis as it may influence the results. However, we found that the group mean correlation between the grasp and load force was ρ=0.26±0.12 (mean and SE), which was not significantly different from zero (one-sample *t* test, *t*_(9)_ = 2.09, *p* > 0.07). Thus, no significant coupling was observed between the grasp force and load force in this experiment.

Next, we examined whether the variance in the reaching movement was different between the wide and narrow conditions. We normalized all trajectories in time and calculated the mean trajectory for each wide and narrow condition using the trials from all three blocks. We then calculated the lateral deviation, defined as the absolute distance halfway into the movement between the cursor’s *x*-axis position and the mean trajectory, for each trial, which is plotted as a function of trials for the population mean in Figure 1*D*. The lateral deviation appeared to be functionally dependent on the width of the visual track. We employed a fit with a linear mixed-effects model (Eq. 1) on the data from all trials, which was labeled by subject, trial number and track condition. A likelihood ratio test revealed a significant effect of the visual track condition on the lateral deviation (χ^2^(2) = 21.95, *p* < 10^−4^). Using the regressed linear model, we calculated the difference in the lateral deviation between the wide and narrow track conditions for the trial range 16–115, i.e., blocks 2–7. A one-sample *t* test on the lateral deviation showed that the subjects’ mean lateral deviation, shown in Figure 1*E*, was significantly smaller in the narrow (0.17 ± 0.02 cm) than the wide (0.23 ± 0.03 cm) condition (*t*_(9)_ = –4.32, *p* < 0.0019), indicating that the subjects’ trajectories were more precise in the narrow condition. What facilitated the subjects’ ability to improve their lateral movement precision in the narrow track?

If the grasp force is correlated with movement precision during reaching, a selective increase in grasp force should be observed in the narrow condition where smaller lateral deviation was observed. The population mean grasp force from each trial is plotted as a function of trials in Figure 1*B*, where the blue and red points are from the wide and narrow conditions, respectively. The average grasp force from a single trial was calculated using data from the entire movement, where the start of the trial was when the target appeared, and the end of the trial was when the hand stopped inside the target. On visual inspection, there appeared to be a functional dependence of the grasp force on the visual track condition and the trial number. The grasp force data from all trials and all subjects were fit with a linear mixed-effects model with the trial number and track condition as predictors (refer to Eq 1). A likelihood ratio test showed that the grasp force was significantly affected by the visual track condition (χ^2^(2) = 118, *p* < 10^−15^). We calculated the mean grasp force in the wide and narrow conditions from the linear model fits, and a one-sample *t* test showed that the grasp force was significantly higher in the narrow than in the wide condition (*t*_(9)_ = 4.41, *p* < 0.0012).

To directly assess the effect of the grasp force on the movement, we plotted the lateral deviation as a function of the grasp force for each subject in the wide (blue) and narrow (red) conditions in Figure 1*F*, with a black line connecting the data from the same subject. Data from each subject were averaged across all three blocks in the wide and narrow conditions to yield one data point per subject per condition. An increase in grasp force was observed to reduce the hand’s lateral deviation, and a non-parametric sign test, which was employed since the data violated normality according to an Anderson–Darling test, found this relationship to be significant (*p* < 0.022). In summary, these results suggest that the grasp force is related to the hand’s movement precision during reaching.

### Experiment 2: divergent force field

In the second experiment, we tested subjects reaching in a divergent force field. The force field applied a force that pushed the hand laterally away from the center line if it deviated laterally from the straight line between the initial and target positions (Fig. 2*A*). The same 10 subjects that took part in the first experiment participated in the divergent force field task (Fig. 2*A*). The trajectories from the first 15 trials inside the divergent force field from a sample subject are plotted in Figure 2*B*. The grasp force, averaged over each trial, is plotted as a function of trials in Figure 2*C*, where the points are the group mean and the shaded area is the SE from all 10 subjects. In the 15 training trials, where subjects reached toward the target without the force field, the grasp force was generally low (Fig. 2*C*, black dots) and continually declined with practice. On trial 16, when the force field was first experienced by subjects, the grasp force was effectively the same as the last training trial. The grasp force increased rapidly in the second and third force field trials, and peaked at approximately the fourth force field trial, after which the grasp force declined exponentially but not to the original level observed in the training trials.

Recall that the second prediction from our hypothesis dictates that increases in the grasp force should only be related to a desired increase in movement precision. Thus, the grasp force should not change even if the actual movement precision increases or decreases. The movement precision in this force field experiment is denoted by the perpendicular distance, defined as the absolute distance from the line at *x* = 0.

First, we examined how the grasp force and the perpendicular distance changed from the last training trial (Fig. 2*D*, dashed trace) to the first force field trial (solid trace). The group mean grasp force and the group mean perpendicular distance are plotted, in Figure 2*D*, as a function of normalized time, where time 0 was the time of target onset and the end was where the subject reached the target. The perpendicular distance was observed to increase dramatically on the first force field trial. The group mean grasp force in the last training trial was 3.1 ± 0.7 N (mean and SE) and for the first force field trial it was 2.9 ± 0.7 N. A paired sample *t* test found that the difference in the grasp force between the last training trial and the first force field trial was not statistically significant (*t*_(9)_ = 2.20, *p* > 0.055). Although the perpendicular distance increased dramatically, the grasp force did not change.

Next, we examined whether the grasp force was different on catch trials in comparison to the force field trials. The group mean grasp force (blue traces) and the perpendicular distance (dashed traces) from all catch trials (dashed traces) and all force field trials (solid traces) is plotted as a function of normalized time. Notably, the grasp force profile is different from the training trial in Figure 2*D*, where it was constant throughout the movement. The grasp force appears to increase in tandem with the perpendicular distance in the force field trials. We found the mean grasp force, taken over the whole trial, and calculated the difference between the mean grasp force in catch trials with the neighboring force field trials. This difference was 0.08 ± 0.12 N, which was statistically not different from zero (one-sample *t* test, *t*_(9)_ = 0.65, *p* > 0.53). Hence, the grasp force did not change in catch trials, although the perpendicular distance clearly decreased.

## Discussion

In this study, we measured the power grasp force while subjects completed two reaching tasks. The first task asked subjects to stay within a visual track during reaching, and the second task had subjects reach to a target while their hand was perturbed by a diverging force field that amplified lateral reaching errors. The results from both experiments support our hypothesis that the grasp force is positively correlated with the desire to increase movement precision. Namely, the grasp force increased when the visual track was narrower and required higher movement precision. Furthermore, the grasp force in the force field did not change in tandem with changes in the actual perpendicular distance, but with the desire to change it.

The latter observation, that the grasp force was not significantly correlated with the load force from the force field, is of importance. Several studies have reported the high correlation between the pinch grip force and the load force from the environment (Flanagan and Wing, 1997; Flanagan et al., 2006). This coupling had to be taken into account when subjects adapted their pinch grip force when reaching in a variable force field (Hadjiosif and Smith, 2015) to avoid the confound where a change in the grip force may be mistaken for an adaptation to the variability of the force field. Instead, the change may have been due to the increased load force from the variable force field. In our experiment, we employed a divergent force field, which has a similar effect to the variable force field used in Hadjiosif and Smith (2015), namely that unpredictable forces are imposed on the subject’s hand that cause movement variability. Unlike the pinch grip force, the power grasp force during reaching was not correlated with the load force from the force field. The power grasp force thus avoids confounds when interpreting changes in the grasp force. However, this may be valid only when the forces from the force field are approximately orthogonal to the placement of the grasp force sensor, and so caution is still required when interpreting the changes in the grasp force.

In both experiments, the grasp force increased from the initial exposure to a visual or force field condition, but gradually decayed as a function of trials. As reported in Hadjiosif and Smith (2015), two opposing adaptation phenomena are likely at play. The first is a fast and sensitive adaptation that increases grasp force to rapidly improve movement precision due to a task demand. The second is a slower adaptation process that optimizes grasp force to conserve effort (Todorov and Jordan, 2002; Emken et al., 2007). The summed contributions of both fast and slow adaptation processes explain why the grasp force increases rapidly from initial exposure, but continually decays throughout our experiment. This decay can be observed in the training trials in both experiments, implying that the gradual decay in the grasp force must be taken into account when interpreting the data. In our first experiment, this was accounted for by the linear mixed-effects model that included a trial gradient. The decay in the grasp force does not contradict our hypothesis, but it shows that an effort conservation process is continually working to reduce excessive grasp force production.

In addition to these adaptation processes, we observed that the increase in grasp force, when switching from a wide to a narrow visual track, was less pronounced in the later trials. Subjects may have learned to update their motion plan to move straighter without having to rely on increasing grasp force to remain inside the visual track (Wong et al., 2009). Such a strategy was likely possible when staying inside the narrow visual track but was infeasible when reaching in the diverging force field that punished even minor lateral deviations. This may explain why the grasp force plateaus to a value above the nominal level observed in the training trials in the divergent force field experiment.

The changes in the grasp force observed when subjects learned to reach in the diverging force field are similar to the results of another study that examined the adaptation of arm cocontraction during the learning of a divergent force field (Franklin et al., 2008). In their study, they also found an initial, rapid increase in cocontraction, followed by a slow and gradual decay, which plateaued above the baseline observed in the training trials. The similarity between the grasp force observed in our study, and the arm cocontraction observed in the study of Franklin et al. (2008), raises the possibility of using the grasp force as a tool to probe the CNS’ desire to improve movement precision in rapid movements, such as during a golf swing (Komi et al., 2008), where the delay introduced by the processing of the electromyography data may be detrimental to the analysis. As such, the grasp force methodology could complement existing methods to measure the cocontraction of muscles to further our understanding of the CNS’ desire to improve movement precision.

## Synthesis

Reviewing Editor: Trevor Drew, University of Montreal Decisions are customarily a result of the Reviewing Editor and the peer reviewers coming together and discussing their recommendations until a consensus is reached. When revisions are invited, a fact-based synthesis statement explaining their decision and outlining what is needed to prepare a revision will be listed below. The following reviewer(s) agreed to reveal their identity: Valeriya Gritsenko.

The study aims at evaluating a relationship between grasp force and movement precision. Two experiments were conducted with the same participants to manipulate lateral deviations of hand trajectory and observe the resulting effect on grasp force. Results broadly show that grasp force increases when tasks are introduced that penalize the perpendicular deviation of the hand from a straight line. High-quality data appear to be generated by this study, however, the analysis and interpretation of these data appear to be lacking. The following major and minor issues were identified.

Major issues

The major issue of this study is its conformational design. The authors should describe in text what alternatives exist to their main hypothesis and what would the rejection of the main hypothesis mean. Results of decreasing grip force with motor learning are largely ignored. These results appear to contradict the hypothesis, this should be explained. The literature on similar motor learning studies with forcefields needs to be included in order to help explain the decreasing stiffness and provide a more general context for the results.

The hypothesis stated at the end of Introduction is not testable as described. The hypothesis should state specifically which relationship is expected and whether it is positive or negative. This will enable the formation of a null hypothesis to test it. The rationale for the statistical analysis of results needs to be provided. The various statistical tests applied to the data need to be put into the context of testing the main hypothesis.

The study is correlational in nature. However, conclusions implying causal relationships are repeatedly made. This needs to be corrected, as it makes the conclusions appear overstated. The rationale and logical reasoning for the “state-dependent model of movement precision” need to be spelled out in more detail. The question is not really of “How might the CNS create a predictive, state-dependent model of movement precision?”, but rather whether it is necessary to explain the data. The authors should strive to provide the simplest possible explanation for the results.

The measures of arm stiffness are not included in the design of the study but widely used as the rationale for study design and interpretation of results. The authors should either choose a different rationale or include measures of stiffness based on their data.

Minor issues

Line 2 and 22: redundant sentences

Line 24: (McGraw, 1940)

Lines 44-45: A hypothesis cannot be confirmed, it can only be rejected. The use of “supporting evidence was provided ...” is suggested.

Lines 51-63: The difficulty of measuring arm stiffness is not a sufficient argument for measuring something else instead. Alternatively, evidence supporting the relationship between grasp force and arm or joint stiffness should be presented. If no such evidence exists, then the comparisons between arm stiffness and grasp force should not be motivating this study.

Line 61: The question prompts the reader to think of alternative strategies. However, no alternative strategies are mentioned besides grasp force.

Line 73: The hypothesis cannot be “validated”. It can only be tested using the scientific method.

Line 83: Why only male subjects were included in the study? Gender bias in experimental results is a serious problem in science.

Lines 136-157: These paragraphs belong in methods. Some information is repeated to that included in the Methods section already.

Fig. 1C: It would be interesting to see the load and grasp force profiles separated for wide and narrow conditions.

Fig. 1D: It appears that the grasp force is continually decreasing throughout the experiment. The increase in grasp force in the narrow condition is most evident in the first time that the narrow condition is introduced (trials 35-45). Then this effect goes away for the end of the experiment (trials 90-105). Does this mean that the grasp force reflects learning a new challenging task in general rather than the limb stiffness in particular?

Fig. 1E: Where along the trajectory were the lateral deviation and grant force measured for this figure? Were they measured at the same time during each trial? What were the error bars across, trials or subjects? Was there a correlation across trials between maximal lateral deviation and the corresponding grip force?

Fig. 1F: The symbols need error bars. The same questions apply to these data as to the data shown in 1E.

Results section: The statistical tests used in this section were not described in Methods. Some parametric and some non-parametric tests appear to have been used. No rationale is provided for either.

Lines 214-222: This belongs in Methods, some information is repetitive.

Lines 259-276: The functions describing the grasp force in Fig. 3A and lateral deviation in Fig. 3B are obviously distinct. The former one reaches a plateau in the first half of the movement, while the latter one increases exponentially. Weak rationale is provided for calculating a linear relationship between them for Fig. 3D.

Fig. 3D the “same-force” correlations vary widely across trials to be meaningful. The “preceding-catch” correlation was calculated differently than the “same-force” one, thus a comparison between them is not possible. Rather than calculating correlations within a trial, the correlations across trials may be more meaningful to explore. The preceding-catch” correlation was calculated over 3 preceding trials, which is an indication of history dependence of grasp force across trials rather than within a trial.

Lines 277-291: It is not clear if repeated measures ANOVA is an appropriate statistical test for the correlation coefficients. Were they normally distributed to warrant a parametric test? Previous analyses used non-parametric tests. The formula for “preceding-catch” correlation was calculated using a different formula than the rest of correlation coefficients. Therefore, they cannot be included in the one-way ANOVA.

Liens 330-335: Insufficient arguments are provided to support this claim.

## Author Response

We thank the Editor and the Reviewer for the care with which they have examined the manuscript. Their comments are addressed below.

REVISIONS

The study aims at evaluating a relationship between grasp force and movement precision. Two experiments were conducted with the same participants to manipulate lateral deviations of hand trajectory and observe the resulting effect on grasp force. Results broadly show that grasp force increases when tasks are introduced that penalize the perpendicular deviation of the hand from a straight line. High-quality data appear to be generated by this study, however, the analysis and interpretation of these data appear to be lacking. The following major and minor issues were identified.

Major issues

The major issue of this study is its conformational design. The authors should describe in text what alternatives exist to their main hypothesis and what would the rejection of the main hypothesis mean. Results of decreasing grip force with motor learning are largely ignored. These results appear to contradict the hypothesis, this should be explained. The literature on similar motor learning studies with forcefields needs to be included in order to help explain the decreasing stiffness and provide a more general context for the results.

The hypothesis stated at the end of Introduction is not testable as described. The hypothesis should state specifically which relationship is expected and whether it is positive or negative. This will enable the formation of a null hypothesis to test it. The rationale for the statistical analysis of results needs to be provided. The various statistical tests applied to the data need to be put into the context of testing the main hypothesis.

We have added two new paragraphs in the Introduction about motor learning in a force field to establish the context of our results, and have changed the rationale of our study, which is to test the hypothesis that the grasp force positively correlates with the desire to increase movement precision. Two predictions based on this hypothesis are tested in the two experiments. These predictions are that the grasp force increases when greater movement precision is demanded from the task, and that increases in the grasp force reflect a desire to improve movement precision, rather than the actual changes in movement precision. If these predictions are false, our hypothesis must be rejected.

The decreasing grasp force is discussed in the Discussion, and how this phenomenon, likely related to effort optimization, does not contradict our hypothesis but is a trend that must be taken into account when interpreting and analysing the grasp force data. The decay in the grasp force is accounted for by the linear mixed-effects model, which has a gradient term that is a function of the trial number. The rationale for the statistical tests has been added to the Methods and they are put into the context of the main hypothesis, as suggested by the reviewer.

The study is correlational in nature. However, conclusions implying causal relationships are repeatedly made. This needs to be corrected, as it makes the conclusions appear overstated. The rationale and logical reasoning for the “state-dependent model of movement precision” need to be spelled out in more detail. The question is not really of “How might the CNS create a predictive, state-dependent model of movement precision?”, but rather whether it is necessary to explain the data. The authors should strive to provide the simplest possible explanation for the results.

We agree with the reviewer that the causal relationship between grasp force and movement precision cannot be determined with our current paradigm. As such, we have removed all mentions of causal links between the two and only mention the positive correlation between the two quantities.

The measures of arm stiffness are not included in the design of the study but widely used as the rationale for study design and interpretation of results. The authors should either choose a different rationale or include measures of stiffness based on their data.

We have modified the rationale to test the hypothesis that the grasp force positively correlates with the desire to improve movement precision. We have included some discussion of the similarity of the adaptation of grasp force in our experiment with what has been reported of arm cocontraction in the literature. However, this is kept to a minimum and is only discussed in the Discussion.

Minor issues

Line 2 and 22: redundant sentences

This has been edited, thank you.

Line 24: (McGraw, 1940)

This citation has been fixed.

Lines 44-45: A hypothesis cannot be confirmed, it can only be rejected. The use of “supporting evidence was provided ...” is suggested.

This change has been made as suggested by the reviewer.

Lines 51-63: The difficulty of measuring arm stiffness is not a sufficient argument for measuring something else instead. Alternatively, evidence supporting the relationship between grasp force and arm or joint stiffness should be presented. If no such evidence exists, then the comparisons between arm stiffness and grasp force should not be motivating this study.

We have modified the rationale of the study to focus on the relationship between increases in the grasp force and the desire to improve movement precision. Two predictions based on this hypothesis are tested in our study.

Line 61: The question prompts the reader to think of alternative strategies. However, no alternative strategies are mentioned besides grasp force.

We have rephrased this sentence.

Line 73: The hypothesis cannot be “validated”. It can only be tested using the scientific method.

This has been changed to “tested”.

Line 83: Why only male subjects were included in the study? Gender bias in experimental results is a serious problem in science.

We did not balance the gender of our subjects as it was not of primary concern in our study.

Lines 136-157: These paragraphs belong in methods. Some information is repeated to that included in the Methods section already.

We have removed this text in the Results.

Fig. 1C: It would be interesting to see the load and grasp force profiles separated for wide and narrow conditions.

The grasp and load forces were separated for the narrow and wide conditions, and are plotted as functions of normalized time in the figure below. Overall, they have similar temporal profiles in terms of shape, with the grasp force during the wide trials having an attenuated profile in comparison to the narrow trials.

Figure A: Grasp and load forces as a function of normalized time, separated between the narrow and wide conditions.

Fig. 1D: It appears that the grasp force is continually decreasing throughout the experiment. The increase in grasp force in the narrow condition is most evident in the first time that the narrow condition is introduced (trials 35-45). Then this effect goes away for the end of the experiment (trials 90-105). Does this mean that the grasp force reflects learning a new challenging task in general rather than the limb stiffness in particular?

Thank you for the comment. We believe that, initially, subjects increase grasp force to move straighter and stay within the visual track. However, increasing grasp force increases effort expenditure. Humans are purported to optimize their movements by minimizing a cost function of task error and effort (Todorov and Jordan, 2002). To conserve effort, subjects may learn to update their motion plan to move straighter, which enables them to slowly decrease grasp force. Alternatively, subjects may learn via reinforcement learning (Wolpert et al., 2001) to slowly reduce unnecessary grasp force, i.e. the “safety margin” of minimum grasp force to stay inside the visual track (Hadjiosif and Smith, 2015). This is discussed in detail in a new paragraph of the Discussion, and how such a decaying trend in the grasp force must be accounted for in the interpretation of the data.

It should be noted that although a decrease in the grasp force was observed in the second experiment of reaching in the divergent force field, the grasp force did not return to the level of the training trials. Instead, it plateaus at a value higher than observed in training trials, indicating that the minimum grasp force needed to reach inside the DF was high, and could not be reduced beyond this level. This is also discussed in a new paragraph of the Discussion.

Fig. 1E: Where along the trajectory were the lateral deviation and grant force measured for this figure? Were they measured at the same time during each trial? What were the error bars across, trials or subjects? Was there a correlation across trials between maximal lateral deviation and the corresponding grip force?

The lateral deviation was measured when the hand was halfway between the initial position and the target position. This point occurred at different times on different trials. The grasp force was averaged across the entire movement, from the onset of the target until the hand stopped inside the target. The deviation in the Narrow condition was 0.17{plus minus}0.02cm and in the Wide condition it was 0.23{plus minus}0.03cm (group mean and standard error); these values have been added to the Results.

We could not find a significant correlation between the lateral deviation and the grasp force across trials. We believe this is due to the decaying grasp force during the experiment that masks the increase in grasp force when the visual track changed from wide to narrow.

Fig. 1F: The symbols need error bars. The same questions apply to these data as to the data shown in 1E.

We have added error bars to the figure. These data are identical to the ones from Figure 1E, but are simply shown as a function of one another (lateral deviation as a function of grasp force) to see whether the negative correlation between lateral deviation and grasp force is observable on a subject level. Figure 1E examined the negative correlation only on a group level.

Results section: The statistical tests used in this section were not described in Methods. Some parametric and some non-parametric tests appear to have been used. No rationale is provided for either.

The rationale for the post-hoc tests has been included in the Methods. The normality of all data sets was examined prior to post-hoc testing. Only one data set violated normality, which is why a non-parametric was used for this data.

Lines 214-222: This belongs in Methods, some information is repetitive.

This has been moved to the Methods.

Lines 259-276: The functions describing the grasp force in Fig. 3A and lateral deviation in Fig. 3B are obviously distinct. The former one reaches a plateau in the first half of the movement, while the latter one increases exponentially. Weak rationale is provided for calculating a linear relationship between them for Fig. 3D.

Thank you for the comment. It was an error to show the grasp force and the perpendicular as a function of the lateral position of the hand. The relationship between the grasp force and the perpendicular distance is comparable when examining their temporal profiles (new Figures 2D and 2E) on the force field and catch trials.

The rationale for comparing the grasp force and the perpendicular distance is that the grasp force increases when there is a desire to improve movement precision. We predicted that the grasp force would remain unchanged if the actual movement precision changes. Thus, the grasp force in the first force field trial and the training trials should be similar, and the grasp force between the force field trials and the catch trials should be similar as well, even though the actual movement precision is quite different.

Fig. 3D the “same-force” correlations vary widely across trials to be meaningful. The “preceding-catch” correlation was calculated differently than the “same-force” one, thus a comparison between them is not possible. Rather than calculating correlations within a trial, the correlations across trials may be more meaningful to explore. The preceding-catch” correlation was calculated over 3 preceding trials, which is an indication of history dependence of grasp force across trials rather than within a trial.

The correlation analysis has been replaced by a comparison of the difference in the grasp force between the last training and the first force field trial, and the difference in the grasp force between all catch trials and neighbouring force field trials. These paired sample t-tests test the prediction that the grasp force responds to desired changes in movement precision, and not the actual changes in movement precision.

Lines 277-291: It is not clear if repeated measures ANOVA is an appropriate statistical test for the correlation coefficients. Were they normally distributed to warrant a parametric test? Previous analyses used non-parametric tests. The formula for “preceding-catch” correlation was calculated using a different formula than the rest of correlation coefficients. Therefore, they cannot be included in the one-way ANOVA.

We have removed the repeated-measures ANOVA as we have simplified our hypothesis to test the correlation between the grasp force and the perpendicular distance in the first force field trial.

Liens 330-335: Insufficient arguments are provided to support this claim.

This paragraph has been removed from the Discussion.
